# Middle-Aged Man With Unstable Angina and an Inaccessible Right Coronary Artery

**DOI:** 10.7759/cureus.11156

**Published:** 2020-10-25

**Authors:** Kameel Kassab, Neeraj Jolly, Aviral Vij

**Affiliations:** 1 Cardiology, John H Stroger, Jr. Hospital of Cook County, Chicago, USA; 2 Cardiology, Rush University Medical Center, Chicago, USA

**Keywords:** acute coronary syndrome, aortic dissection, cardiac computed tomography angiography

## Abstract

Acute myocardial ischemia and infarction from retrograde dissection of the aortic root into the coronary ostia is a potentially fatal condition. Unrecognized type A aortic dissection at the time of angiography for acute coronary syndrome (ACS) carries a high burden of morbidity and mortality. Cardiac computed tomography angiography (CCTA) has emerged as one of the instrumental tools in the diagnosis of retrograde coronary involvement from type A aortic dissections. We present a case of ACS secondary to retrograde aortic dissection extending into the right coronary artery (RCA) suspected during coronary angiography and confirmed by CCTA. The patient was managed surgically with aortic root replacement and coronary artery bypass grafting.

## Introduction

Coronary malperfusion secondary to aortic dissection is a relatively rare syndrome with a reported rate of 7% on post-mortem examination among patients with type A aortic dissection [1]. Initial presentation could be misdiagnosed as an acute coronary syndrome (ACS), leading to delayed diagnosis of aortic dissection. Unrecognized aortic dissection at the time of coronary angiography carries a high burden of morbidity and mortality predominantly secondary to dissection extension by catheter manipulation and the use of anticoagulation [2]. Optimal management usually requires surgical intervention and is dictated by the type of coronary lesion. Perioperative mortality remains high, ranging between 20% and 30% in the reported series [2-4]. 

## Case presentation

**Figure 1:**
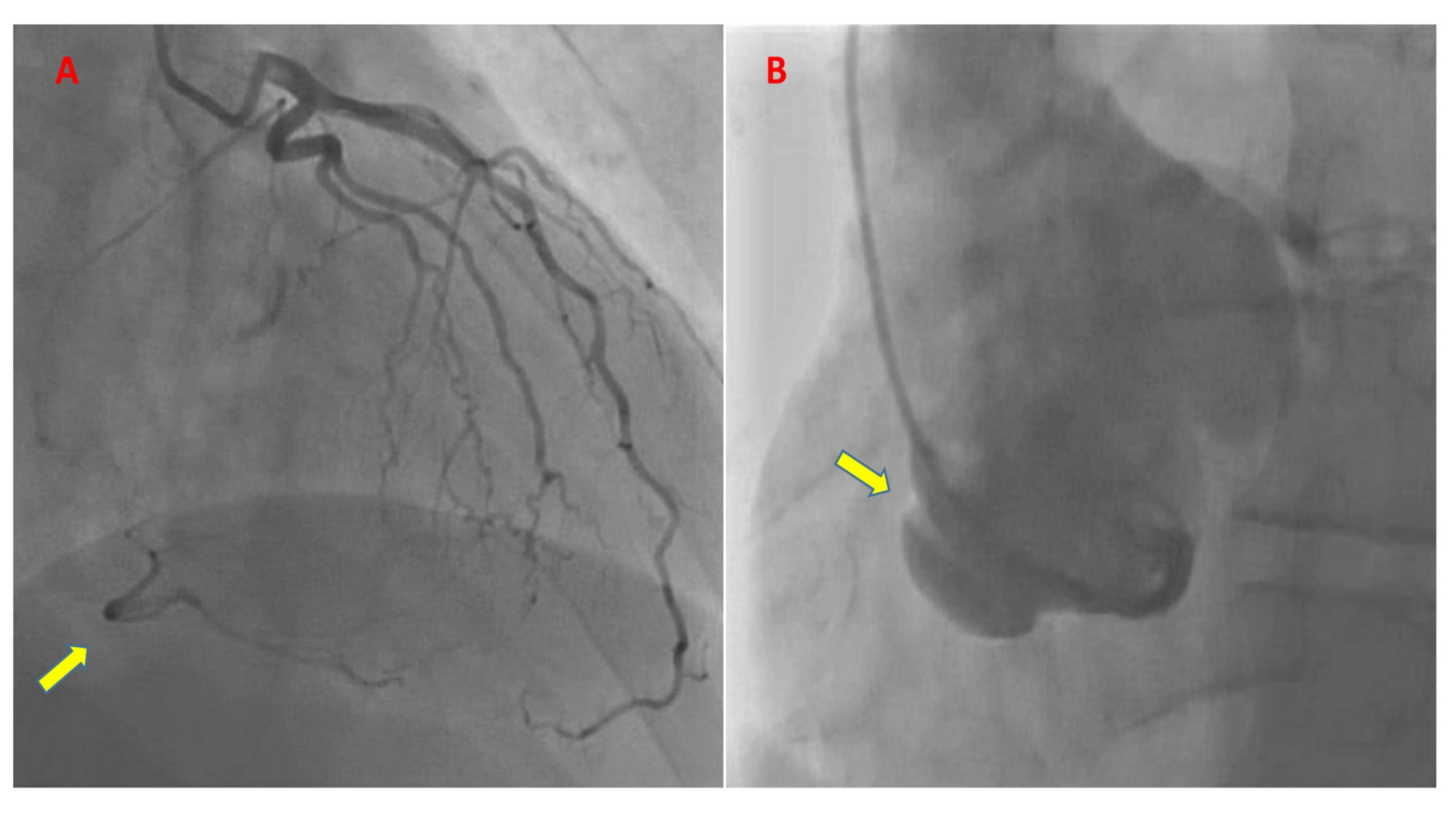
Left coronary angiography with no obstructive coronary artery disease (CAD). (A) Collaterals (arrow) noted filling the right coronary artery in right anterior oblique (RAO) cranial projection. (B) Aortogram in left anterior oblique (LAO) projection showing aneurysmal dilatation of aortic root, absence of opacification of the right coronary artery, and linear hypodensity in the right coronary cusp (arrow) suggestive of dissection.

A 55-year-old man with no significant past medical history presented for evaluation of worsening chest pain. He reported the symptoms of chest pain and heaviness for two weeks prior to presentation, initially on exertion, which later progressed to occur at rest. On presentation, blood pressure was 110/68 mmHg, and heart rate was 73 bpm. Physical examination was within normal limits. Electrocardiogram showed nonspecific T wave inversion in inferior leads. A limited bedside transthoracic echocardiogram showed a normal left ventricular ejection fraction of 55%, hypokinesis of basal to mid inferior myocardium, and a dilated aortic root of 5.3 cm at the level of sinuses of Valsalva with evidence of only mild aortic regurgitation. Laboratory data, including complete blood count and metabolic panel, were unremarkable, and Troponin levels were normal. Given concern for unstable angina, the patient underwent invasive coronary angiogram via a transfemoral route, which showed no evidence of coronary artery disease in the left circulation. Collaterals were noted filling the right coronary artery (RCA) from the left system (Figure 1A). The RCA could not be engaged despite the use of multiple sizes and types of catheters; hence the possibility of “flush” occlusion of the ostium of the RCA was entertained. An aortic root angiography was then performed with a 6 Fr pigtail catheter but failed to show the ostium of RCA or any antegrade flow in the RCA. Additionally, there was a significantly dilated aortic root and an unclear linear hypodensity in the right coronary cusp (Figure 1B). The patient tolerated the procedure well without any remarkable intra-procedural events. 

**Figure 2:**
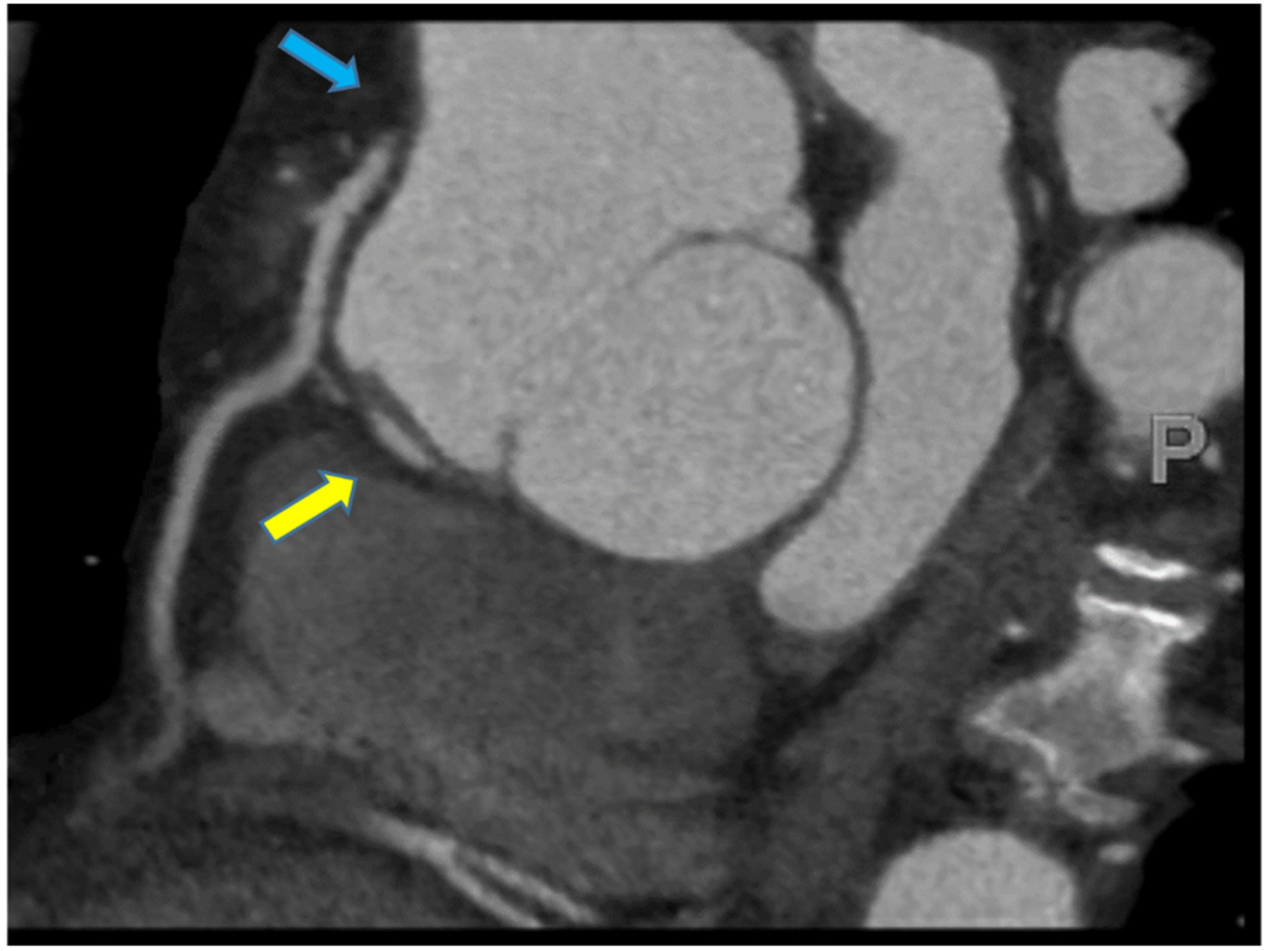
Cardiac computed tomography angiography showing the absence of communication between the ostium of the right coronary artery and the right sinus of Valsalva (blue arrow). Discrete transverse dissection flap visualized in the right sinus of Valsalva (yellow arrow).

Cardiac computed tomography angiography (CCTA) was performed the next day to avoid contrast-induced nephropathy (Figure 2). It revealed a discrete transverse dissection flap visualized in the right sinus of Valsalva, extending 25 mm superiorly. The ostium of the RCA was not communicating with the right coronary cusp. The aortic root was aneurysmal and measured 5.5 cm at the level of sinuses of Valsalva.

The patient was sent for emergent cardiovascular surgery. Cardioplegia was delivered retrogradely through the coronary sinus. Intra-operative findings showed bicuspid aortic valve morphology with aortic root aneurysm. There was complete occlusion of the RCA, and the ostium was noted to be arising from the false lumen of the chronic aortic dissection. The patient underwent aortic root replacement and modified Bentall procedure with a 25 mm St. Jude valve conduit with re-implantation of the left coronary artery and single saphenous coronary artery bypass graft to the right coronary artery. The patient had an unremarkable postoperative course.

## Discussion

Acute myocardial ischemia and infarction due to retrograde dissection of the aortic root reaching the coronary ostia is a potentially fatal condition and has been reported in multiple cohorts [2,4]. Involvement of the right coronary ostium, and hence inferior ischemia, is more common than the involvement of the left coronary system [5]. This latter finding could be related to a survival bias as most patients who dissect into the left coronary circulation may not survive to diagnosis. Most patients will present acutely with chest discomfort; however, some cases may remain asymptomatic.

In describing the acuity of presentation, most type A aortic dissections present acutely; however, a small proportion goes undiagnosed in the acute phase and is found upon delayed presentation of symptoms or incidentally [6]. The clinical presentation of our patient with two weeks of symptoms and the presence of left to right collaterals on invasive angiogram points to a chronic aortic dissection. Chronic type A aortic dissections are older than 14 days. Yet, the subacute designation has also been proposed as between 14 and 90 days [6]. They are significantly more likely to have bicuspid aortic valve morphology and less likely to extend beyond the arch, compared to acute presentations [7]. Chronic cases remain asymptomatic in 69% of the time, with a median of 10 weeks until symptom onset in patients who became symptomatic [6]. This latter finding may explain the delayed presentation of our patient.

Delayed diagnosis of type A aortic dissections, whether acute or chronic, in the setting of ACS may have serious sequela mainly due to the risk of extending the dissection during catheter manipulations. Additionally, anticoagulation during a cardiac catheterization may worsen intramural hematoma and extend the dissection flap [8]. Hence, early recognition and a high index of suspicion are vital prior to any coronary interventions in patients with acute chest syndrome, and chest computed tomography angiography remains the gold standard for diagnosing acute aortic dissections. In our case, ascending aortic dissection was not initially suspected based on the presentation. Several intra-procedural findings, including unusual RCA anatomy, difficulty with coronary engagement, and visualization of possible hypodensity/dissection flap on root angiogram, prompted further evaluation with CCTA for aortic dissection and delineating RCA anatomy/origin. The role of gated CCTA in the diagnosis of coronary extension of aortic dissections has been previously described [9]. In our patient, it was considered the imaging modality of choice as it not only provided the diagnosis of ascending aortic dissection and delineated its extent but also outlined the aortic valve, annular morphology, the aortic root, and coronary anatomy.

Management is surgical. The main surgical issues pertain to optimizing myocardial protection and blood flow restoration to the ischemic myocardium. In the three main surgical series pertaining to the coronary malperfusion syndrome secondary to type A aortic dissection, perioperative mortality was predominantly related to heart failure [2-4]. This latter finding emphasizes the importance of myocardial protection during surgical intervention. Retrograde cardioplegia via the coronary sinus, as utilized in our case, has been advocated for when antegrade flow cannot be established due to ostial coronary occlusion. Antegrade cardioplegia via the uninvolved coronary sinus provides additional myocardial protection [2-4]. The optimal modality for re-establishing flow to the malperfused myocardium has been debated. In an older cohort of 24 patients with coronary ostial dissections, Neri et al. advocated for coronary repair over coronary artery bypass grafting (CABG) [2]. In contrast, Kawahito et al. performed CABG in all of the 12 patients in their cohort to avoid coronary button manipulation [4]. Recently, in a larger cohort of 76 patients, Kreibich et al. advocated for surgical repair in patients with type A lesions (where false lumen involves the coronary ostium) and type B lesions (where false lumen extends into the body of the coronary artery). However, type C lesions (where there is complete avulsion of the coronary artery) usually require CABG to apply antegrade cardioplegia and myocardial protection [3]. Of note, there was no difference in in-hospital mortality among different lesion types.

## Conclusions

Coronary malperfusion secondary to type A aortic dissection is associated with high mortality, with the initial diagnosis of dissection frequently masked as ACS presentation. Early recognition, coronary revascularization, and surgical repair are essential for optimizing clinical outcomes. CCTA is a feasible and progressively more widely available tool in the evaluation of coronary involvement of aortic dissections and has a high diagnostic accuracy for optimal pre-operative planning. 
